# Evaluation of cord blood irisin levels in term newborns with small gestational age and appropriate gestational age

**DOI:** 10.1186/s40064-016-2869-y

**Published:** 2016-10-10

**Authors:** Esengul Keleş, Filiz Fatime Turan

**Affiliations:** 1 Department of Pediatrics, Fatih University, Sahilyolu sk.no: 16 Dragos-Maltepe, 34844 Istanbul, Turkey; 2 Department of Biochemistry, Fatih University, Istanbul, Turkey

**Keywords:** SGA, AGA, Term infant, Cord blood irisin level

## Abstract

**Background:**

Most recently, a novel myokine, named irisin, was identified in human that expressed by skeletal muscle after exercise. Irisin increases energy expenditure by turning white adipose tissue into brown adipose tissue. Thus improves carbohydrate homeostasis in humans. Irisin is considered as a potential biomarker for obesity and metabolic syndrome. In recent years, numerous studies have been conducted about irisin with adults, although number of studies with newborns is limited.

**Objective:**

To evaluate cord blood irisin level with small gestational age (SGA) and appropriate gestational age (AGA) in term newborns.

**Methods:**

A cross-sectional study of 34 AGA and 34 SGA term newborns who were born in (1–30) December 2015 in Fatih University Hospital. Estimated fetal weight were calculated using the Hadlock formula by gynecologists to pregnant women in second trimester. All the babies were classified at birth as SGA or AGA. SGA was defined according to the Lubchenco scale for gender and gestational age. We collected umbilical cord blood at the time of delivery. Cord blood irisin levels were measured using commercial enzyme-linked immunosorbent assays in our hospital laboratory.

**Results:**

Cord blood irisin levels were significantly lower in SGA group [median 30 (25 ± 8) ng/ml] than in AGA group [median 40 (39 ± 13) ng/ml, p < 0.001]. No statistically significant differences were observed among the groups in terms of the demographic features (gender, mode of delivery, gestational weeks, 1–5 min Apgar score) (p > 0.05). Mothers with gestational diabetes, hypertension, asthma, chronic disease, use of drug or a history of smoking exposure were excluded from the study. When the study data were evaluated, Yates Continuity Correction and Fisher’s exact tests were used in descriptive statistical methods and for comparison of qualitative data.

**Conclusion:**

Our results support the idea that irisin have a physiologic role in neonates. Low level of irisin is associated with the impaired carbohydrate metabolism in term infants with SGA. However, further studies with larger series are warranted to confirm this.

## Background

In recent years, the interaction between adipose and muscle tissues has been increasingly recognized to play an important role in body weight regulation and carbohydrate homeostasis. Because the largest organ in the body skeletal muscle accounts for the majority of glucose uptake in response to insulin, and is quantitatively the most important site of insulin resistance. During the past decade, skeletal muscle has also been identified as a secretory organ, secreting myostatin, IL-6, IL-15 and other factors (Pedersen and Febbraio 2012). Most recently, a novel myokine, named irisin, was identified in mice and human that expressed by skeletal muscle type I membrane precursor protein FNDC5 after exercise. It is regulated by peroxisome proliferator-activated receptor-y coactivator-1 (PGC1)-a. Irisin increases energy expenditure by turning white adipose tissue (WAT) into brown adipose tissue (BAT). Thus improves carbohydrate homeostasis in humans (Boström et al. 2012). BAT is the second type of adipose tissue, found primarily in mammals and newborn may also affect insülin sensitivity and whole-body metabolism via its role in termogenesis (Gesta et al. 2007). Recent studies in mice and humans have demonstrated that enhancing brown fat thermogenesis may lead to improved glucose tolerance, increased sensitivity, lower body weight and decreased fat mass (Stanford et al. 2013; Yang et al. 2003). Exogenous administration of irisin using adenoviral delivery triggered a programme of brown-fat-like development in specific depots of WAT, and resulted in increased energy expenditure, improved glucose tolerance, and a modest but significant weight loss (Boström et al. 2012). Changes in the total amount of BAT or a reduction in its thermogenic capacity could therefore be important precursors of later obesity (Rothwell and Stock 1983).

The association between small gestational infant (SGA) and impaired insulin sensitivity with the development of diseases such as type 2 diabetes, cardiovascular diseases or the metabolic syndrome later in life has been demonstrated in several studies (Hofman et al. 1997; Jaquet and Czernichow 2003; Barker 2007). But the underlying mechanisms and the relationship between SGA infants who have lower muscle and BAT tissue, impaired glucose tolerance still remain unclear. There are numerous studies about irisin in adults. However, the physiologic role of irisin in neonates especially in SGA infants remains to be studied.

In this study, we aimed to investigate whether there was a difference between cord levels of irisin between the term infants with SGA and AGA.

## Methods

### Study population

The present study was prospectively conducted to evaluate cord blood irisin level in SGA and AGA infant. Ethics committee approval was received for this study from the ethics committee of Fatih University. The study adheres to the Declaration of Helsinki. Written contents were received from the families of neonates planned to be included in the study. The study included 34 SGA (19 female and 15 male) and 34 AGA (21 female and 13 male) infants who were born in (1–30) December 2015 in Fatih University Hospital.

#### Inclusion criteria

Estimated fetal weight (EFW) were calculated using the Hadlock formula by gynecologists to pregnant women in second trimester in our hospital, which includes abdominal, head circumferences, and femur length measurements (Hadlock et al. 1985).

#### Exclusion criteria

Newborns with fetal or congenital diseases [TORCH infections: Toxoplasmosis, Other (syphilis, varicella-zoster, parvovirus B19), Rubella, Cytomegalovirus (CMV), Herpes infections], congenital malformation, neonatal sepsis and meconium aspiration that could affect fetal growth were excluded. Mothers with gestational diabetes, hypertension, asthma, chronic disease, use of drug or a history of smoking exposure were excluded from the study.

All the babies were classified at birth as SGA or AGA. SGA was defined as birth weight ≤−1.5 SD and AGA >−1.5 SD according to the Lubchenco scala for gender and gestational age (Lubchenco et al. 1963).

### Cord blood collection and measurement of plasma irisin

Umbilical cord blood was collected from the umbilical vein attached to the placenta at the time of delivery. They were centrifuged and the plasma was divided into 0.5 ml aliquots, which were stored in Eppendorf tubes at −80 °C until analysis. Cord blood irisin levels were measured using commercial enzyme-linked immunosorbent assays (ELISA, Phoenix Pharmaceuticals, Inc., Burlingame, CA, USA) in our hospital laboratory. Intra- and inter-assay variances were <4–6 and <8–10 %, and the range of detectable concentration was 0.066–1024 ng/ml.

### Statistical analysis

Statistical analyses were performed using the Statistical Package for Social Sciences-SPSS version 15 software (SPSS Inc., Chicago, Illinois). For categorical variables, the χ^2^ test was used. For group comparisons, the Student t test was used with a normal distribution, and the Mann–Whitney U and Kolmogorov–Smirnov tests with abnormal distributions. Kruskal–Wallis test was used for continuous variables for comparison between the two different birth weight categories (SGA and AGA). Variance analyses and Friedman variance analyses were used for repeated measurements. For descriptive statistics, percent, minimum–maximum–median, mean, and standard deviation were used in accordance with the type and distribution of the variable. Normality of irisin was tested with Shapiro–Wilk test, and the data were transformed to logarithmic scale to obtain normality. A result was considered statistically significant for values of p < 0.05.

## Results

The median birth weight were lower in the SGA (2280 ± 221 g) group compared to AGA (3263 ± 329 g). There was a strongly significant difference between the two groups in terms of birth weight of the babies (p < 0.01). However, no statistically significant differences were observed among the groups in terms of the demographic features (gender, mode of delivery, gestational weeks, 1–5 min Apgar score) (p > 0.05).

**Fig. 1:**
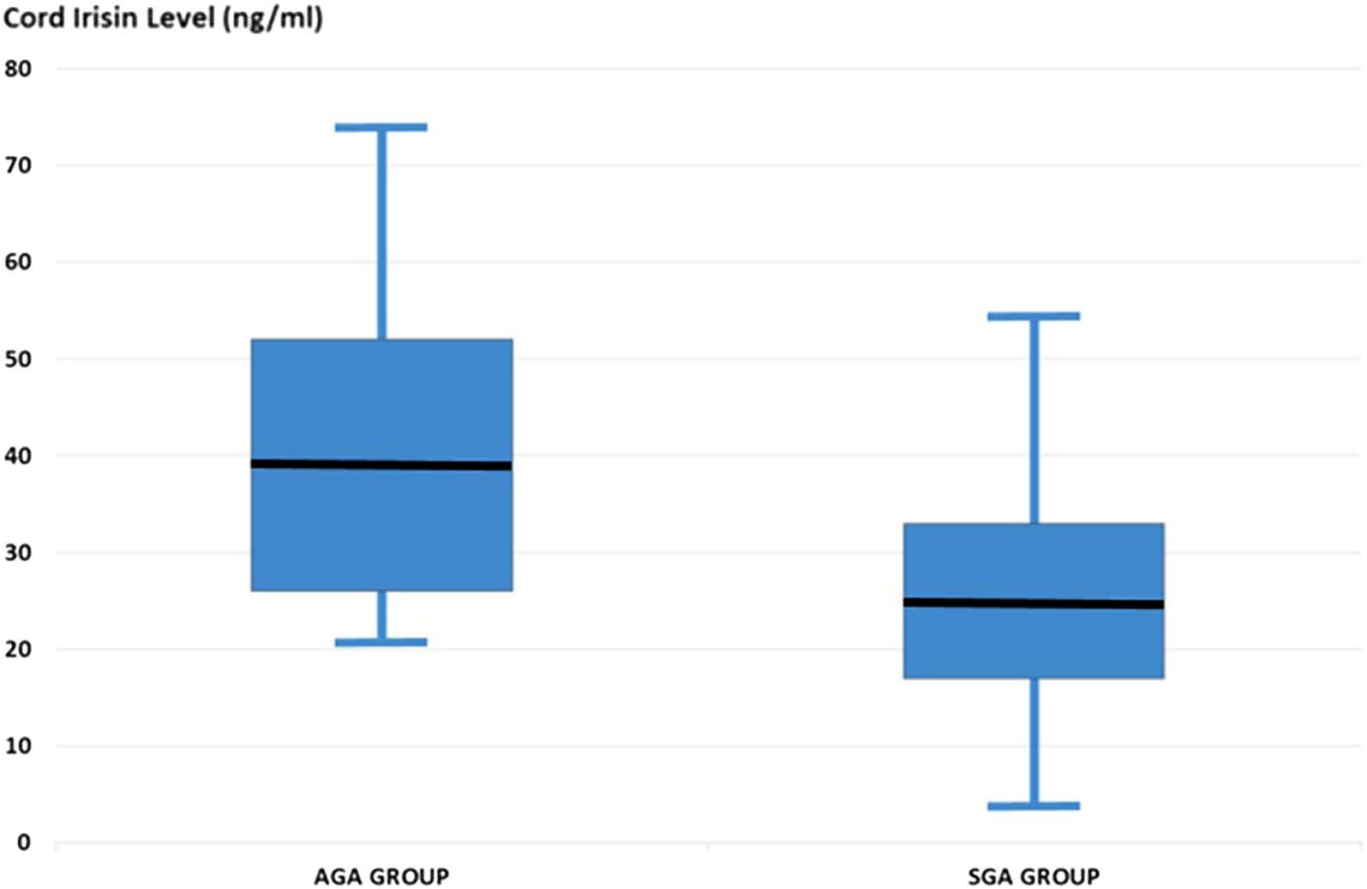
Cord irisin level in AGA and SGA group

But cord blood irisin levels were significantly lower in SGA group [median 30 (25 ± 8) ng/ml] than in AGA group [median 40 (39 ± 13) ng/ml, p < 0.001, Table 1; Fig. 1].

**Table 1:** The demographic characteristics of appropriate for gestational age (AGA) group 1 and small for gestational age (SGA) group 2 infants

	AGA (n = 34)	SGA (n = 34)	Total (n = 68)	p
	Median ± SD	Median ± SD		
Birth weight (g)	3263 ± 329	2280 ± 221	2771 ± 569	^a^0.001*
Gestational age (weeks)	38.9 ± 0.2	38.7 ± 0.5	38.8 ± 0.4	^a^0.108
Apgar score (1th min)	*7*.79 ± 0.76	7.71 ± 0.74	7.75 ± 0.76	^b^0.697
Apgar score (5th min)	8.79 ± 0.72	8.71 ± 0.75	8.75 ± 0.73	^b^0.697
Gender (F/M)	21/13	19/15	40/28	^c^0.768
Cesarean delivery n %	30 (88.2 %)	25 (73.5 %)	55 (80.8 %)	^d^0.461
Mother’s age	30.6 ± 3.8	29.8 ± 3.3	29.6 ± 4.1	^d^0.082
Gravity n %				
1	18 (53 %)	19 (55.9 %)	37 (54.4 %)	^d^0.072
2	6 (17.6 %)	7 (20.6 %)	13 (19.1 %)	
3	10 (29.4 %)	8 (23.5 %)	18 (26.5 %)	
Irisin (ng/ml)				
Median ± SD	39 ± 13	25 ± 8	32 ± 13	^b^0.001*
Min–max	21–74	14–52	25–63	

* *p* < 0.01

^a^Student t test

^b^Mann Whitney U test

^c^Yates Continuity Correction

^d^Fisher’s exact test

There was not a statistically significant difference between the groups in the number of conceptions and gestational age (p > 0.05). Mothers with gestational diabetes, hypertension, asthma, chronic disease, use of drug or a history of smoking exposure were excluded from the study. Multivariate linear regression analysis was not carried out, because the pregnants carrying maternal risk factors that could affect irisin level.

## Discussion

Our prospective study shows that cord blood irisin levels are positively correlated with birth weight in term infants and the levels are decreased in SGA compared to AGA infant. Today number of the studies about serum levels of irisin in term infants with SGA and AGA is limited. We believe that it would provide contribution to the literature.

Because the underlying mechanisms and the relationship between SGA infants who have lower muscle and BAT tissue, impaired glucose tolerance still remain unclear. Also irisin is considered as a potential biomarker for obesity and metabolic syndrome.

Studies from the literature have usually emphasized the excess nutrition during early life include resetting of hypothalamic energy sensing and appetite regulation, altered adipose tissue insulin sensitivity and impaired BAT function (Stettler et al. 2002; Druet et al. 2012; Soto et al. 2003; Shalini et al. 2013). Also in animal models it has been demonstrated that in utero malnutrition affects pancreatic β-cell development leading to impaired β-cell function later in life (Garofano et al. 1997, 1999; Limesand et al. 2005). However this studies have shown that excess nutrition is a risk factor for obesity and insulin resistance not only in SGA infants but also in AGA neonates. For example; in a large multicentre randomized trial of protein supplementation of infant formula milk in 1138 infants, those receiving the higher-protein milk formula (albeit containing less protein than contemporary commercial formula milks) were of increased bodyweight at 2 years of age (Koletzko et al. 2009).

Today, different physiopathological factors are more commonly mentioned in explanation of impaired carbohydrate metabolism which is seen more frequently in SGA cases and the studies have focused particularly on irisin that is seen as a potential biomarker for development of obesity and metabolic syndrome. In the most recent study, Joung et al. found plasma irisin levels were positively correlated with gestational age (r = 0.21, p < 0.001), and birth weight Z-score (r = 0.18, p < 0.001) (Joung et al. 2015). In that study, there was a significant difference between the gestational age of newborns (26–41 GW), whereas in our study, the mean gestational week was found as GW = 38.8 ± 0.4. Though it is stated in the same publication that, no significant correlation was found between the gestational week and irisin level in multivariate analysis (β = 0.005, p = 0.43), this result may be attributed to that, 257 cases were AGA and 60 were SGA newborns, because there is a numerically significant difference between the groups. In order to eliminate this difference, we equated the number of subjects in both groups (AGA = 34, SGA = 34). Again in the same report, no statistically significant difference was found between GW and irisin in contrary to the hypothesis of body mass index and BAT are correlated with irisine, which is emphasized in the same study (SGA, AGA, LGA median 55.38, 64.41, 68.70 ng/ml; respectively). Because BMI and BAT of premature infants are low, independently from term newborns being SGA, AGA or LGA. In our study, in order to eliminate this difference all newborns enrolled were chosen from term infants (median 38.8 ± 0.4 GW). Furthermore, it is reported in the mentioned study that, singleton infants of mothers with preeclampsia had lower cord blood irisin levels compared to infants of mothers with our preeclampsia. Unlike that study, pregnants with the conditions that could affect irisin level such as maternal preaclampsia, gestational diabetes, asthma, chronic disease or a history of drug use were excluded from our study.

In addition our study, mothers with gestational diabetes, hypertension, asthma, chronic disease, use of drug or a history of smoking exposure were not included, because Garces et al. recently reported that circulating irisin levels in pregnant women with preeclampsia were lower compared to women with healthy pregnancies in the third trimester (Garces et al. 2014). Additionally, recent reports of irisin in relation to gestational diabetes mellitus (GDM) have shown controversial results between maternal circulating irisin levels and GDM (Yuksel et al. 2014; Ebert et al. 2014).

Studies in the literature conducted with healthy adults found higher irisin levels unlike our study (Park et al., n = 107 ng/ml) (Park et al. 2013). Whereas in the present study we found significantly lower irisin levels in infants compared to those in adults [median 30 (32 ± 13) ng/ml]. This difference might be attributable to the smaller muscular mass in neonates. Because in human tissues, the distribution of FNDC5 expression was strongly increased in muscle in comparison with adipose tissue, similar to the findings described in mice (Huh et al. 2012). Huh et al. found, age-related muscle loss correlated to decreased circulating irisin concentration, muscle mass being the main predictor of this in humans. Furthermore after bariatric surgery-induced weight loss, circulating irisin levels as well as muscle FNDC5 gene expression were significantly down-regulated (Huh et al. 2012).

I think that, likewise the study by Joung et al., lower cord irisin levels found SGA infants in our study might be influenced by the smaller muscular mass as well as lower BAT (Joung et al. 2015). Because, José et al. found a positive association of FNDC5 gene expression with BAT markers (PRDM16 and UCP1), lipogenic (FASN and ACC), and the expression of insulin-pathway related genes (GLUT4 and IRS1), mitochondrial (MTCO3 and PGC1), and alternative macrophage markers (IL-10 and CD206) (José et al. 2013). Also van Marken Lichtenbelt et al. showed that the amount of BAT was significantly decreased in association with obesity, with a negative linear relationship between BAT, BMI, and percent body fat (van Marken Lichtenbelt et al. 2009).

Based on all these studies and the present study; I have thought that irisin which is recently rather emphasized for its association with insulin resistance and obesity and released by muscle tissue, regulating glucose homeostasis by influencing the BAT might be a physiopathological factor in explanation of the impaired carbohydrate metabolism which is more common in SGA infants. Because both muscular mass and BAT are low in SGA infants.

## Conclusion

Low levels of irisin in term infants with SGA may be a factor in explanation of the impaired carbohydrate metabolism in these infants. However, further studies with larger series are warranted to confirm this.
